# Pseudo-merohedral twinning and noncrystallographic symmetry in orthorhombic crystals of SIVmac239 Nef core domain bound to different-length TCRζ fragments

**DOI:** 10.1107/S090744490904880X

**Published:** 2010-01-22

**Authors:** Walter M. Kim, Alexander B. Sigalov, Lawrence J. Stern

**Affiliations:** a University of Massachusetts Medical School, USA

**Keywords:** pseudo-merohedral twinning, noncrystallographic symmetry, pseudosymmetry, human immunodeficiency virus, Nef, T-cell receptor

## Abstract

HIV/SIV Nef mediates many cellular processes through interactions with various cytoplasmic and membrane-associated host proteins, including the signalling ζ subunit of the T-­cell receptor (TCRζ). Here, the crystallization strategy, methods and refinement procedures used to solve the structures of the core domain of the SIVmac239 isolate of Nef (Nef_core_) in complex with two different TCRζ fragments are described. The structure of SIVmac239 Nef_core_ bound to the longer TCRζ polypeptide (Leu51–Asp93) was determined to 3.7 Å resolution (*R*_work_ = 28.7%) in the tetragonal space group *P*4_3_2_1_2. The structure of SIVmac239 Nef_core_ in complex with the shorter TCRζ polypeptide (Ala63–Arg80) was determined to 2.05 Å resolution (*R*_work_ = 17.0%), but only after the detection of nearly perfect pseudo-merohedral crystal twinning and proper assignment of the orthorhombic space group *P*2_1_2_1_2_1_. The reduction in crystal space-group symmetry induced by the truncated TCRζ polypeptide appears to be caused by the rearrangement of crystal-contact hydrogen-bonding networks and the substitution of crystallographic symmetry operations by similar noncrystallographic symmetry (NCS) operations. The combination of NCS rotations that were nearly parallel to the twin operation (*k*, *h*, −*l*) and *a* and *b* unit-cell parameters that were nearly identical predisposed the *P*2_1_2_1_2_1_ crystal form to pseudo-merohedral twinning.

## Abstract 2

*P*2_1_2_1_2_1_ crystals of SIV Nef core domain bound to a peptide fragment of the T-cell receptor ζ subunit exhibited noncrystallographic symmetry and nearly perfect pseudo-merohedral twinning simulating tetragonal symmetry. For a different peptide fragment, nontwinned tetragonal crystals were observed but diffracted to lower resolution. The structure was determined after assignment of the top molecular-replacement solutions to various twin or NCS domains followed by refinement under the appropriate twin law.

## 1. Introduction

Protein crystallization occurs under supersaturating conditions where protein molecules organize by either noncrystallographic or crystallographic symmetry operations into repeating unit cells that pack to form a crystal lattice. Crystal twinning occurs when two or more crystal packings intersperse in one larger aggregate crystal. This has been reported to occur as a result of polymorphic transformation during physical stress (Yeates, 1997 ▶; Govindasamy *et al.*, 2004 ▶), but more commonly occurs as a pathology of crystal growth. When the lattices of each crystal packing in the aggregate crystal do not overlap in three dimensions, the crystal exhibits epitaxial, or nonmerohedral, twinning, which can easily be detected by the presence of split reflections in the crystal’s X-ray diffraction pattern. However, when the lattice axes of the individual crystals are parallel the crystal is considered to be mero­hedrally twinned and the X-ray diffraction pattern will not provide any visual cues of crystal twinning. For protein molecules, merohedrally twinned crystals exist predominately as hemihedrally twinned crystals (Yeates, 1997 ▶) which contain two distinct twin domains that are related to each other by a twin-law operation. The twin fraction α represents the fractional contribution of the less prevalent twin domain. The diffraction pattern of a hemihedrally twinned crystal is therefore the superimposition of two unique diffraction patterns, one from each twin domain, where each reflection intensity is the weighted sum of two twin-related intensities (Grainger, 1969 ▶), 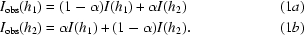
The individual intensities *I*(*h*_1_) and *I*(*h*_2_) can be solved by combining the linear equations
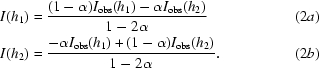
As the twin fraction α approaches 1/2 the crystal is considered to be perfectly twinned and calculation of the intensities *I*(*h*_1_) and *I*(*h*_2_) begins to fail as the term (1 − 2α) begins to approach zero. This complicates the process of twin-related reflection intensity calculation, commonly referred to as detwinning. Structure determination has therefore preferentially been performed for hemihedral crystals that exhibit nonperfect twinning.

Less common cases of twinning have been described where a twin-law operation supports a higher Laue symmetry than that of the crystal unit cell (Rudolph *et al.*, 2004 ▶). This type of twinning, which is referred to as pseudo-merohedral twinning, can occur in special circumstances such as a monoclinic system where the β angle approaches 90° (Larsen *et al.*, 2002 ▶) or an orthorhombic system where the unit-cell axes *a* and *b* are fortuitously similar in length (*a* ≃ *b*), resulting in the emulation of higher apparent tetragonal symmetry (Brooks *et al.*, 2008 ▶). In this report, we describe such a case for crystals of a complex of the Nef (negative factor) protein from simian immunodeficiency virus bound to a fragment of one of Nef’s cellular targets, the cytosolic domain of the TCR ζ subunit (TCRζ).

Nef from human immunodeficiency virus (HIV) or simian immunodeficiency virus (SIV) is a 27–35 kDa viral accessory protein that is dispensable for replication but required for high infectivity and virulence (reviewed in Arien & Verhasselt, 2008 ▶). Expressed in abundance early in the viral life cycle, Nef performs a number of functions that can be generalized into three activities: enhancement of viral infectivity, downregulation of surface receptors and modulation of T-cell activation. Notable among Nef’s functions is the interaction of Nef with TCRζ (Sigalov *et al.*, 2008 ▶; Fackler *et al.*, 2001 ▶; Swigut *et al.*, 2000 ▶; Schaefer *et al.*, 2000 ▶; Xu *et al.*, 1999 ▶; Howe *et al.*, 1998 ▶; Bell *et al.*, 1998 ▶), the principal signaling component of the T-cell antigen receptor. This interaction has been suggested to play a role in HIV-mediated modulation of membrane-proximal T-cell signaling events (Fenard *et al.*, 2005 ▶; Thoulouze *et al.*, 2006 ▶) and in SIV-mediated downregulation of the T-cell receptor (Schindler *et al.*, 2006 ▶; Swigut *et al.*, 2003 ▶; Schaefer *et al.*, 2002 ▶; Munch *et al.*, 2002 ▶; Willard-Gallo *et al.*, 2001 ▶). In previous work (Schaefer *et al.*, 2000 ▶), SIV and HIV-2 Nef have been shown to bind TCRζ at two unique sites denoted ‘SIV Nef interaction domains’ (SNIDs), the first containing elements of immunoreceptor tyrosine activation motif (ITAM) 1 and the second containing elements of ITAM 2. However, the structural features of Nef that determine its specificity for TCRζ remain unknown.

Nef contains two domains: an unstructured highly variable myristylated N-terminal domain and a C-terminal structured core domain (Arold *et al.*, 1997 ▶; Grzesiek *et al.*, 1997 ▶; Lee *et al.*, 1996 ▶) that exhibits high sequence conservation among different HIV-1, HIV-2 and SIV isolates. The Nef conserved core domain (Nef_core_) has been described to be responsible for the majority of Nef’s interactions (Peter, 1998 ▶), including the TCRζ-binding activity of SIV Nef. The core domain of HIV-1 Nef has been shown to be amenable to crystallization (Arold *et al.*, 1997 ▶; Lee *et al.*, 1996 ▶; Franken *et al.*, 1997 ▶). In this study, we aimed to determine the crystal structure of the Nef–TCRζ complex by crystallizing complexes of SIVmac239 Nef_core_ with TCRζ fragment polypeptides containing the putative binding regions.

Here, we describe the crystallization and structure determination of the complexes of SIVmac239 Nef_core_ with two different TCRζ polypeptides, TCRζ_DP1_ and TCRζ_A63–R80_. Structure determination of the SIVmac239 Nef_core_–TCRζ_DP1_ complex was hampered by the poor electron-density maps calculated from the low-resolution diffraction data and phases derived from molecular replacement using the published HIV Nef core-domain structures. Eventually, we were able to determine this structure using the higher resolution SIVmac239 Nef_core_–TCRζ_A63–R80_ complex as a starting model. However, determination of the high-resolution SIVmac239 Nef_core_–TCRζ_A63–R80_ complex structure was hindered by the nearly perfect pseudo-merohedral crystal twinning that was detected on analysis of the intensity statistics. Ultimately, a partially twinned crystal with a twin fraction of 0.426 was used to solve the structure of the SIVmac239 Nef_core_–TCRζ_A63–R80_ complex to 2.05 Å resolution. The structures of the two complexes revealed that crystallization of SIVmac239 Nef_core_ with the shorter TCRζ polypeptide had reduced the space-group symmetry from tetragonal to orthorhombic and introduced noncrystallographic symmetry (NCS). Because the unit-cell axes *a* and *b* were still nearly identical in the orthorhombic crystal form, the crystal was prone to twinning. This study presents a unique case in which pseudo-merohedral crystal twinning is the consequence of a reduction in crystal symmetry induced by the truncation of a protein ligand.

## 2. Materials and methods

### 2.1. Protein expression and purification

The core domain of SIVmac239 Nef with a two-residue linker, GS-Nef (Asp95–Ser235) (Nef_core_), was expressed and purified as described by Sigalov *et al.* (2008 ▶). Briefly, Nef was expressed as a 6×His-thioredoxin fusion protein in *Escherichia coli* BL21 (DE3) cells. Following cell lysis, the Nef fusion protein was isolated by Ni–NTA affinity chromatography (Qiagen) under denaturing conditions (8 *M* urea) and then dialyzed against a nondenaturing buffer containing 20 m*M* Tris, 150 m*M* NaCl, 100 µ*M* DTT at pH 8.0. The soluble fusion protein was then subjected to proteolysis with thrombin (MP Biochemicals), resulting in the cleaved Nef_core_ protein. SIVmac239 Nef_core_ was purified by anion-exchange and size-exclusion chromatography and concentrated to 700 µ*M* by ultrafiltration (Amicon) in PBS.

TCRζ_cyt_ includes an acid-labile Asp-Pro sequence (Landon, 1977 ▶) at positions 93–94. We made use of this to prepare two fragments of TCRζ_cyt_, termed DP1 (Leu51–Asp93) and DP2 (Pro94–Arg164). TCRζ_cyt_, purified as described by Sigalov *et al.* (2004 ▶), was incubated at 1.3 mg ml^−1^ (0.1 m*M*) in 30% acetonitrile, 0.5%(*v*/*v*) TFA for 48 h at 323 K. Fragments were isolated by reverse-phase chromatography on a Vydac C18 300 Å pore-size column using an acetonitrile gradient in 0.1% TFA and recovered by lyophilization. Mass spectrometry was used to verify the identity of the fragments and the lack of any additional chemical modification other than the desired amide hydrolysis. All TCRζ polypeptides were solubilized in 20 m*M* Tris pH 8.0 to a final concentration of 700 µ*M*. Full-length TCRζ was expressed and purified as reported previously (Sigalov *et al.*, 2004 ▶).

### 2.2. Crystallization

Crystals of SIVmac239 Nef_core_ in complex with the TCRζ polypeptides TCRζ_DP1_ and TCRζ_A63–R80_ were grown at 277 K using the hanging-drop vapor-diffusion method (McPherson, 1982 ▶). Crystals of the Nef_core_–TCRζ_DP1_ complex were grown in a 2 µl hanging drop by mixing 0.5 µl SIVmac239 Nef_core_ (700 µ*M*), 0.5 µl TCRζ_DP1_ (700 µ*M*) and 1 µl crystallization buffer (15% PEG 3350, 150 m*M* KF, 100 m*M* HEPES pH 8.2). Crystals of the Nef_core_–TCRζ_A63–R80_ complex were grown in a 3 µl hanging drop by mixing 1 µl SIVmac239 Nef_core_ (700 µ*M*), 1 µl TCRζ_A63–R80_ (700 µ*M*) and 1 µl crystallization buffer (10–15% PEG 3350, 200 m*M* NH_4_F, 100 m*M* HEPES pH 7.4–7.5). The hanging drops were suspended on siliconized glass cover slips over 1 ml crystallization buffer in 24-well plates (Vydax). Crystals of the Nef_core_–TCRζ polypeptide complexes grew to maximal size (750 × 150 × 150 µm) in 3–7 d. Prior to X-ray diffraction experiments, the Nef_core_–TCRζ_DP1_ and Nef_core_–TCRζ_A63–R80_ crystals were transferred into cryoprotectant solutions containing 20–25% ethylene glycol in their respective crystallization buffers and then flash-cooled in liquid nitrogen.

### 2.3. Data collection and processing

16 data sets were collected from various Nef_core_–TCRζ crystals, of which three were used for structure determination. One low-resolution data set (3.7 Å) for the SIVmac239 Nef_core_–TCRζ_DP1_ complex and two high-resolution data sets (1.9 and 2.05 Å) for the SIVmac239 Nef_core_–TCRζ_A63–R80_ complex were collected from single crystals at the National Synchrotron Light Source (beamline X29) using an ADSC Quantum-315r CCD detector system. The crystal-to-detector distances for the Nef_core_–TCRζ_DP1_ complex and the Nef_core_–TCRζ_A63–R80_ complex crystals were 275 and 250 mm, respectively. The crystals were exposed for 1 s with an oscillation of 1° per image. A total of 180 images were collected for each data set, which were separately indexed, integrated and scaled in *HKL*-2000 (Otwinowski & Minor, 1997 ▶). Detection and analysis of crystal twinning was performed in *phenix.xtriage* from the *PHENIX* software package (Adams *et al.*, 2002 ▶). Determination of the twin law governing the pseudo-merohedrally twinned SIVmac239 Nef_core_–TCRζ_A63–R80_ crystals was performed following proper assignment of the crystal space group as described below.

### 2.4. Structure determination and refinement

The structures of the SIVmac239 Nef_core_–TCRζ_A63–R80_ and SIVmac239 Nef_core_–TCRζ_DP1_ complexes were determined by molecular replacement. Firstly, the atomic coordinates of the Nef core domain from HIV-1 were extracted from the crystal structures of the unliganded HIV-1 isolate LAI Nef structure (PDB code 1avv; Arold *et al.*, 1997 ▶), the HIV-1 isolate LAI Nef–Fyn SH3 domain complex (PDB code 1avz; Arold *et al.*, 1997 ▶) and HIV-1 isolate NL4-3 Nef–Fyn SH3 (R96I) domain complex (PDB code 1efn; Lee *et al.*, 1996 ▶) and were modified with *CHAINSAW* (Stein, 2008 ▶) to trim the side chains not shared by SIVmac239 Nef (44% sequence identity) to methyl groups. The modified Nef coordinate sets were used as an ensemble search model for molecular replacement in *Phaser* (Storoni *et al.*, 2004 ▶) and single solutions with translation-function *Z* scores (TFZ) greater than 6.0 were used as starting models. The structure of the Nef_core_–TCRζ_A63–R80_ complex was solved by multiple rounds of twinned refinement using *phenix.refine* from the *PHENIX* software package (Adams *et al.*, 2002 ▶) interspersed with rounds of manual model building and fitting of *F*_o_ − *F*_c_ and 2*F*_o_ − *F*_c_ electron-density maps in *Coot* (Emsley & Cowtan, 2004 ▶). The twin operator (*k*, *h*, −*l*) was applied during each round of refinement, which included three cycles of individual atomic displacement factor refinement and individual energy-minimization procedures accompanied by refinement of the twin fraction α. Water molecules were added to the refined model using both *phenix.refine* and *Coot*. The quality of the final refined SIVmac239 Nef_core_–TCRζ_A63–R80_ structure was validated in *PROCHECK* (Las­kowski *et al.*, 1993 ▶).

The structure of the refined SIVmac239 Nef_core_–TCRζ_A63–R80_ complex was used as a search model to find a molecular-replacement solution for the SIVmac239 Nef_core_–TCRζ_DP1_ data. A single top molecular-replacement solution (TFZ = 10.5, LLG = 266) was found and used as a starting model. The structure of the SIVmac239 Nef_core_–TCRζ_DP1_ structure was solved by refinement consisting of several rounds of individual atomic displacement factor refinement and individual energy-minimization procedures using *phenix.refine*. Model inspection was performed between each round of refinement and the model was modified in *Coot*. The final refined structure was validated for acceptable chemical properties with *PRO­CHECK*. Final model and refinement statistics for both SIVmac239 Nef_core_–TCRζ polypeptide structures are shown in Table 1 ▶ and Ramachandran plots generated by *RAMPAGE* (Lovell *et al.*, 2003 ▶) are provided in Supplementary Figs. S1 and S2^1^.

## 3. Results and discussion

### 3.1. Crystallization and data collection of two SIVmac239 Nef_core_–TCRζ polypeptide complexes

In order to determine the structure of the Nef–TCRζ complex, mixtures of the structured core domain of SIVmac239 Nef (Asp95–Ser235) with various polypeptides spanning the putative binding regions of TCRζ (Schaefer *et al.*, 2000 ▶) were screened for crystal formation. Initial crystallization experiments were aimed towards crystallizing the complex of Nef_core_ (SIVmac239, HIV-1 ELI and NL4-3) with the full-length cytoplasmic domain of TCRζ (TCRζ_cyt_), but these were unsuccessful. Therefore, a polypeptide crystallization screening strategy was employed to identify a minimal TCRζ poly­peptide that bound SIVmac239 Nef_core_, which exhibited the highest affinity TCRζ_cyt_ binding among the SIV, HIV-1 and HIV-2 variants tested (SIVmac239, HIV-1 ELI and NL4-3, and HIV-2 ST; unpublished results).

**Figure 1:**
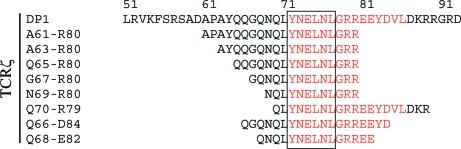
TCRζ polypeptide crystallization screen. The polypeptide sequences are shown with residue position numbers assigned on the left. The boxed region contains the sequence of the first of the two reported SIV Nef interaction domains (Schaefer *et al.*, 2000 ▶). The sequence of ITAM 1 is colored red.

Crystallization efforts focused on TCRζ fragments that contained the N-terminal of the two SIV-interaction domains (Fig. 1 ▶). Of a series of polypeptides spanning TCRζ_cyt_, a peptide included in this region, TCRζ_A61–R80_, bound to Nef_core_ from HIV-1, HIV-2 and SIV strains (manuscript in preparation) and the structural information obtained for the higher affinity SIV variant might be relevant for HIV-1 as well as the more homologous HIV-2 Nef proteins. Moreover, TCRζ_DP1_ (*i.e.* the N-terminal acid-cleavage fragment, residues Leu51–Asp93) formed a 1:1 stoichiometric complex with SIVmac239 Nef_core_ (unpublished results), as did intact TCRζ_cyt_ (Sigalov *et al.*, 2008 ▶). Therefore, a series of polypeptides containing the original TCRζ_DP1_ polypeptide, the shorter TCRζ_A61–R80_ polypeptide and several peptides containing the proposed SNID-1 (Schaefer *et al.*, 2000 ▶) sequence were either prepared from full-length TCRζ_cyt_ (TCRζ_DP1_) or chemically synthesized (TCRζ_A61–R80_ and variants) and used in crystallization experiments with SIVmac239 Nef_core_.

**Figure 2:**
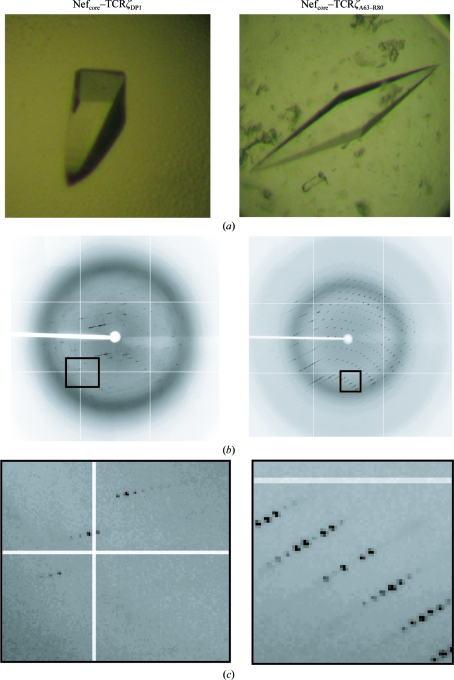
Crystallization and diffraction. (*a*) Crystals of the SIVmac239 Nef_core_–TCRζ_DP1_ and SIVmac239 Nef_core_–TCRζ_A63–R80_ complexes. Both crystals grew to 750 × 150 × 150 µm at 277 K. (*b*) Diffraction patterns of crystals of the SIVmac239 Nef_core_–TCRζ polypeptide complexes collected on beamline X29 at the National Light Synchrotron Light Source, Brookhaven National Laboratory. (*c*) Enlarged view of the diffraction patterns. The diffraction-pattern spot profiles are singular, with no evidence of split spots.

SIVmac239 Nef_core_ crystallized in complex with TCRζ_DP1_ and TCRζ_A63–R80_ under similar conditions. Crystals of the SIVmac239 Nef_core_–TCRζ_DP1_ complex grew readily as long tetragonal pyramids (Fig. 2 ▶ *a*) but diffracted X-rays to low resolution (3.7 Å; Fig. 2 ▶ *b*) and could not be improved further by optimization of the crystallization conditions. In contrast, crystals of SIVmac239 Nef_core_ bound to TCRζ_A63–R80_ adopted a bipyramidal shape (Fig. 2 ▶ *a*) and diffracted X-rays to high resolution (1.9–2.05 Å; Fig. 2 ▶ *b*). Neither crystal form exhibited the concave or ‘re-entrant’ features that have been suggested to predict the presence of twinned crystals (Yeates, 1997 ▶), nor did their diffraction patterns contain split reflections (Fig. 2 ▶ *c*).

### 3.2. Space-group determination and molecular replacement for SIVmac239 Nef_core_–TCRζ_DP1_

The low-resolution diffraction data for the SIVmac239 Nef_core_–TCRζ_DP1_ complex were indexed in the tetragonal Laue group 4/*mmm* (422 point group), with unit-cell parameters *a* = *b* = 51.638, *c* = 189.449 Å and an *R*_merge_ of 5.1%. The Matthews coefficient *V*_M_ (Matthews, 1968 ▶) was calculated to be 2.86 Å^3^ Da^−1^ (57.1% solvent), indicating the presence of one SIVmac239 Nef_core_–TCRζ_DP1_ heterodimer per asymmetric unit. After analysis of the *h*00, 0*k*0 and 00*l* reflection intensities, the space group was further assigned as *P*4_3_2_1_2 or *P*4_1_2_1_2 based on the indicated presence of screw axes along *a* and *c*. Using an ensemble of HIV-1 Nef core domain structures as a search model, a single molecular-replacement solution (TFZ = 9.4, LLG = 63) was found in space group *P*4_3_2_1_2. However, model refinement and building were hindered by the poor quality of the σ_A_-weighted *F*_o_ − *F*_c_ and 2*F*_o_ − *F*_c_ electron-density maps, resulting in an *R*_free_ that could not be reduced below 41%.

### 3.3. Initial space-group determination and molecular replacement for SIVmac239 Nef_core_–TCRζ_A63–R80_

Two crystals of SIVmac239 Nef_core_ bound to the shorter TCRζ_A63–R80_ polypeptide diffracted X-rays to higher resolution (1.9–2.05 Å), but space-group determination proved to be more complicated than for the *P*4_3_2_1_2 crystal form of Nef_core_–TCRζ_DP1_ described above. The diffraction patterns that were observed for Nef_core_–TCRζ_A63–R80_ appeared to be consistent with the lattice previously observed for the lower resolution Nef_core_–TCRζ_DP1_ crystals, and the TCRζ_A63–R80_ data (crystal 1) were initially indexed in the same tetragonal Laue group 4/*mmm* (422 point group), with unit-cell parameters *a* = *b* = 47.203, *c* = 182.939 Å. The integration statistics were similar to those observed previously (*R*_merge_ = 7.6%). Visual inspection of reflection intensities using *HKLVIEW* (Collaborative Computational Project, Num­ber 4, 1994 ▶) and evalution of the *R*_merge_ and Δ*I*/σ(*I*) statistics confirmed the presence of each of the symmetry elements comprising the 422 point group. All of the unit-cell parameters were reduced by 4–10% compared with the SIVmac239 Nef_core_–TCRζ_DP1_ crystals, which is potentially consistent with the shorter length of the TCRζ polypeptide ligand. Analysis of the *h*00 and 0*k*0 intensities indicated the presence of 2_1_ screw axes along *a* and *b*. However, 00*l* intensities were observed for *l* = 2*n*, which is consistent with a 4_2_ (or 2_1_) axis along *c* but inconsistent with a 4_3_ axis as observed for the lower resolution tetragonal SIVmac239 Nef_core_–TCRζ_DP1_ data. A search for a molecular-replacement solution for the SIVmac239 Nef_core_–TCRζ_A63–R80_ data in 4_2_2_1_2 or any of the other 422 space groups yielded no solutions. A data set that was collected from a second SIVmac239 Nef_core_–TCRζ_A63–R80_ crystal (crystal 2) resulted in similar difficulties with molecular replacement. The ambiguous space-group assignment and the inability to find a molecular-replacement solution for the SIVmac239 Nef_core_–TCRζ_A63–R80_ complex in the tetragonal Laue symmetry group suggested the possibility of crystal twinning and required space-group re-evaluation.

### 3.4. Detection and analysis of twinning

**Figure 3:**
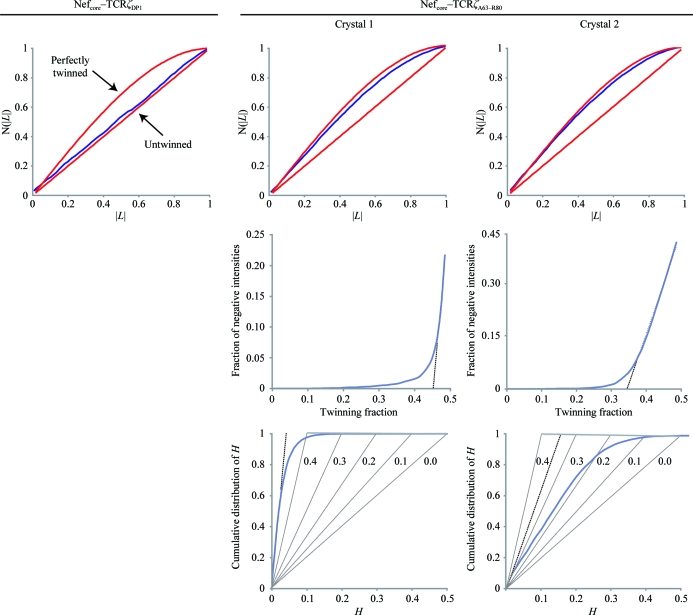
Detection of twinning and estimation of the twin fraction α. Top row, cumulative intensity difference plot of the intensity difference of local pairs of intensities that are not twin-related |*L*| {*L* = [*I*(*h*_1_) − *I*(*h*_2_)]/[*I*(*h*_1_) + *I*(*h*_2_)]} against the cumulative probability distribution *N*(*L*) of the parameter *L* (Padilla & Yeates, 2003 ▶). The expected plots for untwinned and twinned acentric data (red) and the calculated plots for the SIVmac239 Nef_core_–TCRζ polypeptide data (blue) are shown. Middle row, estimation of the twin fraction α by Britton plot analysis (Britton, 1972 ▶). The percentage of negative intensities after detwinning is plotted as a function of the assumed value of α. Overestimation of the twin factor α results in an increase in the percentage of negative intensities. The estimated value of α is extrapolated from the linear fit (dashed line). Bottom row, estimation of the twin fraction α using the *H* plot (Yeates, 1988 ▶). The cumulative fractional intensity difference of acentric twin-related intensities *H* {*H* = |*I*(*h*_1_) − *I*(*h*_2_)|/[*I*(*h*_1_) + *I*(*h*_2_)]} is plotted against *H*. The initial slope (dashed line) of the distribution is a measure of α. The expected slopes for the indicated twin fractions 0.0–0.4 are shown (dotted lines).

A number of statistical methods have been developed to characterize crystal twinning, including the recently developed Padilla–Yeates algorithm for detection of the presence of crystal twinning (Padilla & Yeates, 2003 ▶) and the Britton plot for estimation of the twin fraction α (Britton, 1972 ▶). To assess the twinning of the SIVmac239 Nef_core_–TCRζ polypeptide crystals, several analyses of the intensity statistics were per­formed in *phenix.xtriage*. Firstly, the second moments of the intensities of acentric data (〈*I* ^2^〉/〈|*I*|^2^〉) were calculated for all three SIVmac239 Nef_core_–TCRζ polypeptide complexes. Un­twinned and twinned data are expected to have 〈*I* ^2^〉/〈|*I*|^2^〉 values of 2.0 and 1.5, respectively. The SIVmac239 Nef_core_–TCRζ_DP1_ crystal had an 〈*I* ^2^〉/〈|*I*|^2^〉 value of 2.106, suggesting the absence of twinning, whereas the SIVmac239 Nef_core_–TCRζ_A63–R80_ crystals had 〈*I* ^2^〉/〈|*I*|^2^〉 values of 1.676 (crystal 1) and 1.628 (crystal 2), indicating the presence of twinning in both crystals. A more robust method of twin detection that uses cumulative local intensity deviation distribution statistics as determined by the Padilla–Yeates algorithm (Padilla & Yeates, 2003 ▶) was also employed. In a plot of the local intensity difference |*L*| of non-twin-related intensities *versus* the distribution of the local intensity differences *N*|*L*|, the presence of twinning can be deduced by comparing the experimental plots with the expected plots for twinned and untwinned data (Padilla & Yeates, 2003 ▶). The SIVmac239 Nef_core_–TCRζ_DP1_ data plot was linear, which is consistent with the expected curve for untwinned data (Fig. 3 ▶, top). In contrast, the plots for both SIVmac239 Nef_core_–TCRζ_A63–R80_ crystals 1 and 2 were curved, suggesting the presence of crystal twinning (Fig. 3 ▶, top). The *L* test, which is also based on the local intensity differences of non-twin-related reflection pairs, was additionally employed in order to confirm twinning in the SIVmac239 Nef_core_–TCRζ_A63–R80_ crystals; for untwinned data |*L*| and mean *L* ^2^ are expected to be 1/2 and 1/3, respectively, and for twinned data they are expected to be 3/8 and 1/5, respectively. The SIVmac239 Nef_core_–TCRζ_DP1_ data had calculated |*L*| and *L* ^2^ values of 0.473 and 0.307, which were consistent with an absence of appreciable twinning. The SIVmac239 Nef_core_–TCRζ_A63–R80_ crystals had calculated |*L*| and *L* ^2^ values of 0.402 and 0.229 for crystal 1 and 0.390 and 0.218 for crystal 2, further supporting the presence of crystal twinning. All of the twinning tests suggested that the low-resolution SIVmac239 Nef_core_–TCRζ_DP1_ crystal was not appreciably twinned, whereas the high-resolution SIVmac239 Nef_core_–TCRζ_A63–R80_ crystals were pseudo-merohedrally twinned with a twin fraction near 0.5.

In order to estimate the twin fraction α in the two pseudo-merohedrally twinned SIVmac239 Nef_core_–TCRζ_A63–R80_ crystals, Britton plot (Britton, 1972 ▶) and *H*-plot (Yeates, 1988 ▶) analyses were performed (Fig. 3 ▶, middle and bottom). Crystal 1 exhibited near-perfect twinning, with an estimated twin fraction of 0.452 from the Britton plot and of 0.477 from the *H* plot. In contrast, crystal 2 seemed to be only partially twinned, with estimated twin fractions of 0.344 and 0.356 from the Britton plot and *H* plot, respectively. These initial estimates of the twin fraction based on statistical analysis of intensities were found to underestimate the actual twin fraction, which refined upwards during structure determination to 0.500 and 0.426 for crystals 1 and 2, respectively (see below).

Because the Laue group 4/*mmm* does not support merohedral twinning, we explored the possibility that the twinned SIVmac239 Nef_core_–TCRζ_A63–R80_ crystals were orthorhombic crystals that emulated tetragonal symmetry owing to pseudo-merohedral crystal twinning. The twinned SIVmac239 Nef_core_–TCRζ_A63–R80_ diffraction data were therefore re-indexed in the orthorhombic point group 222. The unit-cell parameters *a* and *b* that are constrained to be equal in point group 422 (Laue group 4/*mmm*) refined to slightly different values of *a* = 47.197 and *b* = 47.208 Å for crystal 1 and *a* = 47.417 and *b* = 47.421 Å for crystal 2, with no significant changes in *R*_merge_ values (Table 1 ▶). Inspection of the *h*00, 0*k*0 and 00*l* intensities indicated the presence of 2_1_ screw axes along each axis, suggesting a *P*2_1_2_1_2_1_ crystal space group. The Matthews coefficient *V*_M_ (Matthews, 1968 ▶) was calculated to be 2.64 Å^3^ Da^−1^ (53.42% solvent) for crystal 1 and 2.64 Å^3^ Da^−1^ (53.99% solvent) for crystal 2, which is consistent with two SIVmac239 Nef_core_–TCRζ_A63–R80_ heterodimers comprising the asymmetric unit.

**Figure 4:**
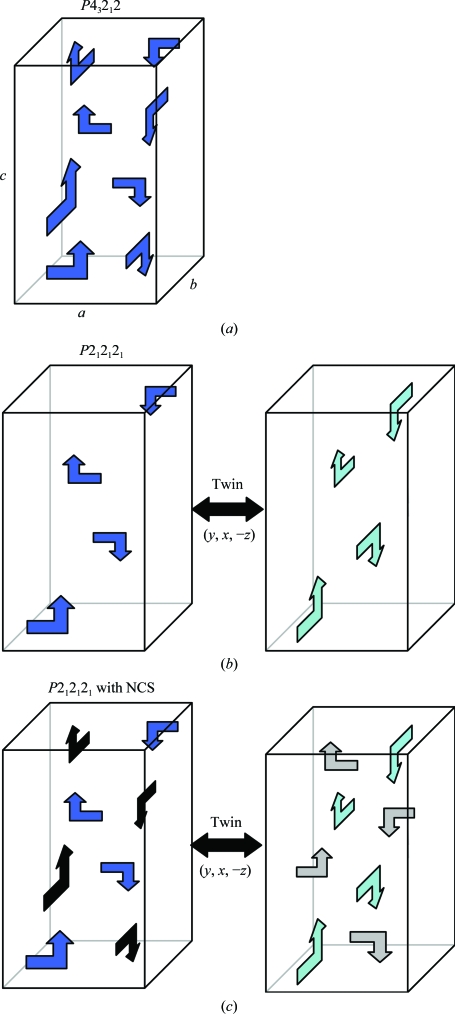
Twinning in an orthorhombic *P*2_1_2_1_2_1_ crystal. (*a*) A *P*4_3_2_1_2 space-group unit cell with one molecule (arrow) per ASU (eight per unit cell) is shown with axes *a*, *b* and *c* labeled. (*b*) A *P*2_1_2_1_2_1_ space-group unit cell with one molecule per ASU (four per unit cell) is shown (left) with its twin unit cell (right) related by the twin operator (*y*, *x*, −*z*). (*c*) A *P*2_1_2_1_2_1_ space-group unit cell with two molecules per ASU (four per unit cell) is shown (left) with its twin unit cell (right) related by the twin operator (*y*, *x*, −*z*). The ASU is comprised of one blue and one black arrow related by noncrystallographic symmetry.

The reduction in symmetry from a fourfold axis along *c* in the tetragonal unit cell to a twofold axis in the orthorhombic unit cell, together with an increase in the number of molecules per asymmetric unit, helped us to identify the pseudo-merohedral twin operation (*k*, *h*, −*l*) that accounted for the apparent fourfold Laue symmetry observed in the diffraction data. Consider a *P*2_1_2_1_2_1_ unit cell with unit-cell length *a* approximately equal to unit-cell length *b* (Fig. 4 ▶). Pseudo-merohedral twinning can exchange the *a* and *b* axis under the twin relationship (*h*, *k*, *l*)→(*k*, *h*, −*l*), resulting in apparent tetragonal symmetry around the *c* axis (Fig. 4*b* ▶). The apparent symmetry observed in this case will be indistinguishable from a nontwinned *P*4_3_2_1_2 (or *P*4_1_2_1_2) unit cell (Fig. 4*a* ▶), of which *P*2_1_2_1_2_1_ is a subgroup. Note that in this case the twinned *P*2_1_2_1_2_1_ unit cell is less tightly packed, with one molecule per asymmetric unit (four per unit cell), than the corresponding nontwinned tetragonal *P*4_3_2_1_2 (or *P*4_1_2_1_2) cell, with one molecule per asymmetric unit (eight per unit cell). Based on the Matthews coefficient, we expected two molecules per asymmetric unit for the twinned *P*2_1_2_1_2_1_ unit cell. Note that the nontwinned crystals of the SIVmac239 Nef_core_–TCRζ_DP1_ complex, which did adopt true tetragonal symmetry with unit-cell parameters similar to those of the twinned *P*2_1_2_1_2_1_ crystal, had a Matthews coefficient consistent with one molecule per asymmetric unit. Because the SIVmac239 Nef_core_–TCRζ_DP1_ and SIVmac239 Nef_core_–TCRζ_A63–R80_ complexes had similar molecular sizes and crystallized in related unit cells with similar lengths and angles we expected similar packing, but this was inconsistent with the different packing expected for the related twinned *P*2_1_2_1_2_1_ and nontwinned *P*4_3_2_1_2 (or *P*4_1_2_1_2) unit cells shown in Fig. 4 ▶. Noncystallographic symmetry relationships that are similar to crystallographic symmetry operators also can result in observed symmetry that is higher than that actually present in the crystal. For example, breakdown of the crystallographic fourfold axis in a tetragonal cell could result in an orthorhombic cell with pseudo-fourfold symmetry. In this case, the noncrystallographic symmetry relationship is similar to the missing crystallographic operator and the related tetragonal and orthorhombic unit cells would have similar packing (Fig. 4*c* ▶). This arrangement can be particularly prone to pseudo-merohedral twinning as a result of the similarity of the crystal packing along the *a* and *b* unit-cell axes (Fig. 4*c* ▶). We explored this scenario as an explanation for the observed twinning in the *P*2_1_2_1_2_1_ crystals with packing similar to nontwinned *P*4_3_2_1_2 (or *P*4_1_2_1_2) crystals.

### 3.5. Structure determination and refinement of SIVmac239 Nef_core_–TCRζ_A63–R80_ using twinned data

**Figure 5:**
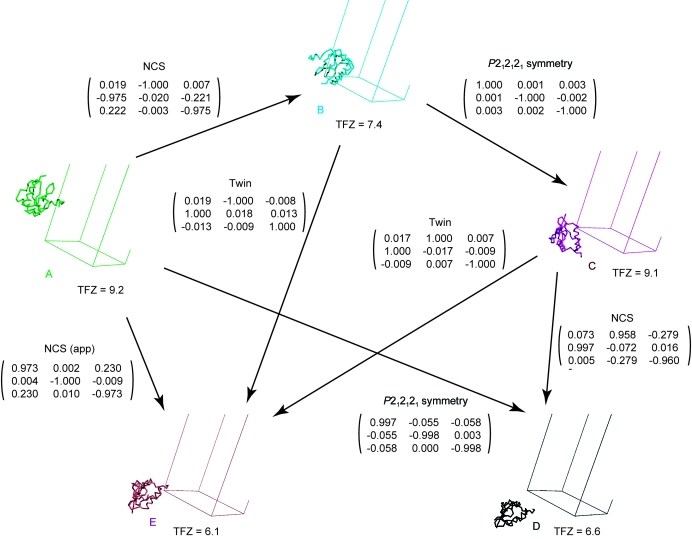
Molecular-replacement solutions. Five molecular-replacement solutions (A–E) are shown with their translation-function *Z* (TFZ) scores denoted. The relationships and rotation matrices relating the molecular-replacement solutions are shown.

After assignment of the SIVmac239 Nef_core_–TCRζ_A63–R80_ crystal data to the orthorhombic *P*2_1_2_1_2_1_ space group, several strong molecular-replacement solutions were readily found with TFZ scores of 6.1–9.2 using a consensus model derived from unliganded HIV-1 Nef_core_ crystal structures. In principle, molecular-replacement solutions corresponding to both twin orientations and noncrystallographically related molecules are expected. Transformations among these solutions were examined to assign each to a twin or NCS domain (Fig. 5 ▶). All solutions could be accounted for using a single NCS transformation, the (*k*, *h*, −*l*) twinning operator and the *P*2_1_2_1_2_1_ space-group symmetry. Two nearby molecules (*A* and  *B*) in the same twin domain related by an approximate 90° rotation were selected to comprise the asymmetric unit.

**Figure 6:**
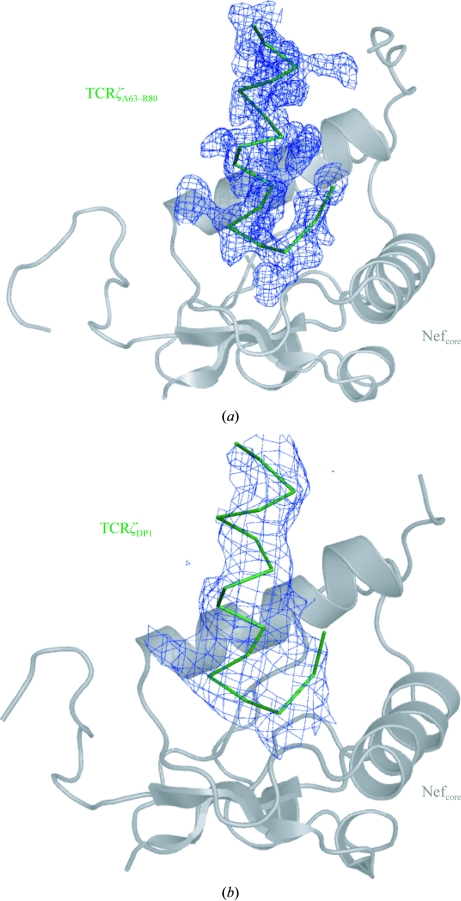
2*F*_o_ − *F*_c_ OMIT electron-density maps of the TCRζ polypeptide. 2*F*_o_ − *F*_c_ OMIT electron-density maps contoured at 1σ calculated from the detwinned *P*2_1_2_1_2_1_ data of the SIVmac239 Nef_core_–TCRζ_A63–R80_ crystal (*a*) and the *P*4_3_2_1_2 data of the SIVmac239 Nef_core_–TCRζ_DP1_ crystal (*b*) are shown for the region encompassing the TCRζ polypeptide.

Once the twinning arrangement was properly understood and taken into account, model building and refinement in space group *P*2_1_2_1_2_1_ with two molecules per asymmetric unit was relatively straightforward. The twin operation (*k*, *h*, −*l*) was factored into each round of twinned refinement in *phenix.refine*, which included three cycles of individual atomic displacement parameter (*B* factor) and energy-minimization refinement. The twin fraction α was also refined in each round and used to detwin the intensity data in order to generate interpretable 2*F*_o_ − *F*_c_ and *F*_o_ − *F*_c_ OMIT electron-density maps suitable for manual model building. However, during the first round of refinement, the twin fraction converged to 0.5 for crystal 1 and 0.426 for crystal 2. The calculated 2*F*_o_ − *F*_c_ electron-density maps generated by *phenix.refine* were noticeably less interpretable for crystal 1 than for crystal 2. Therefore, structure determination proceeded with crystal 2 through iterative cycles of twinned refinement interspersed with rounds of model inspection and building. As the model and twin fraction continued to be refined, there was a marked improvement in the quality of the electron-density maps that allowed the building of five additional residues at the N-­terminus (Val98–Val102), one residue at the C-terminus (Gly234) and 11 residues in the internal disordered loop (Pro197–Trp207) of Nef; the starting model generated from the published crystal structures of HIV-1 Nef was missing nine residues at the N-terminus, two residues at the C-terminus and 29 residues in the disordered loop. Clear density for the TCRζ_A63–R80_ polypeptide ligand was observed and this region was also built into the structure, with 13 of the 16 resolved residues comprising a canonical α-helix (Fig. 6 ▶). Water molecules were added to the model using the automated water-picking functions in *phenix.refine* and *Coot*. The final structure (crystal 2) contained 120 residues of SIVmac239 Nef, 16 residues of TCRζ, 116 ordered water molecules and had *R*_work_ and *R*_free_ values of 17.0% and 18.4%, respectively (Table 1 ▶).

### 3.6. Structure determination and refinement of SIVmac239 Nef_core_–TCRζ_DP1_

With a high-resolution structure for SIVmac239 Nef_core_–TCRζ_A63–R80_ structure in hand, we returned to the low-resolution nontwinned SIVmac239 Nef_core_–TCRζ_DP1_ *P*4_3_2_1_2 data set. In order to determine the low-resolution structure of SIVmac239 Nef_core_–TCRζ_DP1_, the high-resolution SIVmac239 Nef_core_–TCRζ_A63–R80_ structure was used as the starting model for refinement and building. Structure determination by molecular replacement was repeated for the nontwinned SIVmac239 Nef_core_–TCRζ_DP1_ data. A stronger molecular-replacement solution was found (TFZ = 10.5, LLG = 266) but in the same general orientation as that described previously. Refinement of atomic positions and individual *B* factors was performed in *phenix.refine* as described above for the twinned crystal, although without the twin-refinement and detwinning steps. The TCRζ_DP1_ peptide extends 12 residues futher at the N-terminus and 14 residues further at the C-­terminus compared with the TCRζ_A63–R80_ polypeptide, but no additional electron density was observed beyond that seen in the SIVmac239 Nef_core_–TCRζ_A63–R80_ complex (Fig. 6 ▶), suggesting that both the N- and C-termini of the TCRζ_DP1_ fragment were disordered and that no additional Nef contacts were present. The final structure of the SIVmac239 Nef_core_–TCRζ_DP1_ complex contained 111 residues of Nef and 16 residues of TCRζ and had *R*_work_ and *R*_free_ values of 30.1% and 32.9%, respectively (Table 1).

### 3.7. Analysis of the *P*2_1_2_1_2_1_ and *P*4_3_2_1_2 crystal forms of the SIVmac239 Nef_core_–TCRζ polypeptide complex

**Figure 7:**
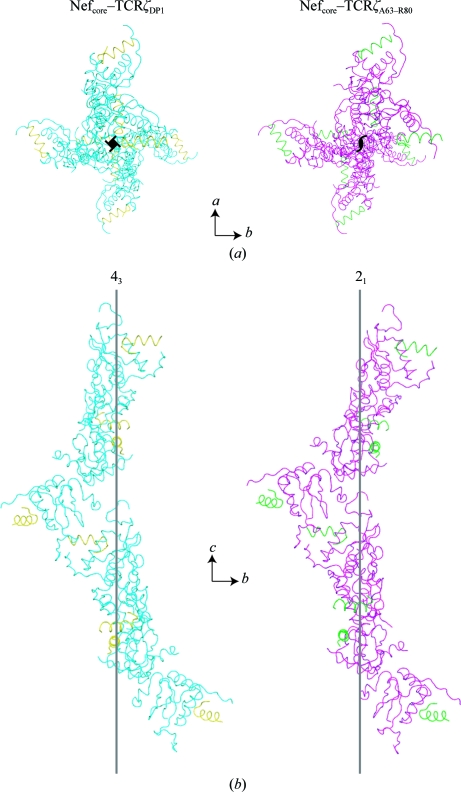
Crystal packing of the *P*4_3_2_1_2 and *P*2_1_2_1_2_1_ crystal forms. (*a*) The crystal symmetry organization of the *P*4_3_2_1_2 crystal form (left) and the *P*2_1_2_1_2_1_ crystal form (right) is shown viewed down the fourfold symmetry axis and the corresponding twofold symmetry axis for the two SIVmac239 Nef_core_–TCRζ polypeptide complexes. In (*b*) the crystal packing along the *c* axis is shown for both crystal forms. SIVmac239 Nef_core_ and TCRζ are colored cyan and yellow (left) and magenta and green (right), respectively.

As described above, the SIVmac239 Nef_core_–TCRζ polypeptide complex crystallized in two related but different crystal lattices depending on the length of the TCRζ ligand. In the presence of the longer 43-residue TCRζ_DP1_ polypeptide SIVmac239 Nef_core_ crystallized in the tetragonal *P*4_3_2_1_2 space group with one SIVmac239 Nef_core_–TCRζ_DP1_ heterodimer comprising the asymmetric unit. In the presence of the shorter 18-residue TCRζ_A63–R80_ polypeptide the complex unexpectedly crystallized in the *P*2_1_2_1_2_1_ space group with severe pseudo-hemihedral twinning. In this crystal form, a rotation axis parallel to *c* exhibited pseudo-fourfold symmetry that deviated slightly from the crystallographic fourfold screw axis observed in the *P*4_3_2_1_2 crystal form (Fig. 7 ▶). The overall packing of the unit cell was also condensed in the ortho­rhombic crystal form, as evidenced by an ∼4 Å (∼8%) reduction in the *a* and *b* axes and an ∼6 Å (∼3%) shortening of the *c* axis.

**Figure 8:**
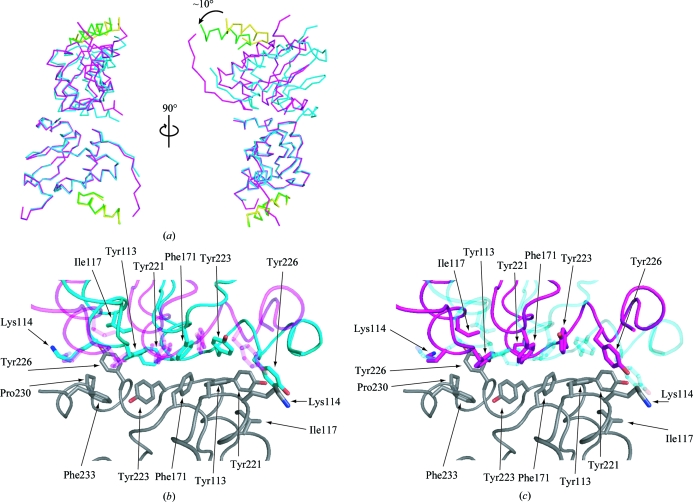
SIVmac239 Nef_core_ dimer interface in the *P*4_3_2_1_2 and *P*2_1_2_1_2_1_ crystal forms. (*a*) Overlay of the two molecules in the asymmetric unit of the *P*2_1_2_1_2_1_ crystal (magenta/green) and two symmetry-related molecules [(*x*, *y*, *z*), (*y*, *x*, −*z*)] in the *P*4_3_2_1_2 crystal (cyan/yellow). The SIVmac239 Nef_core_ is colored magenta (*P*2_1_2_1_2_1_ crystal) or cyan (*P*4_3_2_1_2 crystal) and the TCRζ polypeptide is colored green (*P*2_1_2_1_2_1_ crystal) or yellow (*P*4_3_2_1_2 crystal). The structures of the lower SIVmac239 Nef_core_–TCRζ polypeptide complexes were aligned by least-squares methods. The relative 10° counterclockwise rotation of the top *P*2_1_2_1_2_1_ crystal SIVmac239 Nef_core_–TCRζ polypeptide complex is depicted. (*b*, *c*) Detailed view of the SIVmac239 Nef_core_ dimer interface in the *P*2_1_2_1_2_1_ (magenta) and *P*4_3_2_1_2 (blue) crystals. The aligned lower SIVmac239 Nef_core_–TCRζ polypeptide complex is colored grey and the side chains of residues involved in the interface are shown as stick models. (*b*) *P*2_1_2_1_2_1_ crystal form (cyan) is highlighted. (*c*) *P*4_3_2_1_2 crystal form (magenta) is highlighted.

The transformation from the tetragonal to the ortho­rhombic crystal system was caused by the introduction of noncrystallographic symmetry (NCS) and rearrangement of the hydrogen-bonding network at the crystal contact sites. The *P*4_3_2_1_2 crystal form contained one molecule per asymmetric unit. The *P*2_1_2_1_2_1_ crystal form contained two molecules per asymmetric unit which were no longer related by a crystallo­graphic twofold symmetry operation (*y*, *x*, −*z*) but instead by a twofold NCS operation,
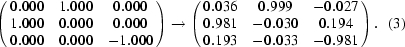
In the tetragonal crystal form the SIVmac239 Nef_core_–TCRζ_DP1_ complex and its symmetry-related partner (*y*, *x*, −*z*) form an antiparallel dimer similar to the crystallographic dimer described previously for the HIV-1 Nef_core_ (Arold *et al.*, 1997 ▶). Structural alignment of one SIVmac239 Nef_core_–TCRζ_A63–R80_ complex from the *P*2_1_2_1_2_1_ crystal form with its corresponding molecule in the *P*4_3_2_1_2 crystallographic dimer reveals that the NCS-related molecule in the orthorhombic crystal form is rotated by ∼10° from its corresponding molecule in the *P*4_3_2_1_2 crystal form (Fig. 8 ▶). The interface between the two molecules involves the C-terminus of SIVmac239 Nef_core_ and is predominantly occupied by aromatic residues (Tyr113, Tyr221, Phe171, Tyr223 and Tyr226). As shown in Fig. 8 ▶(*b*), SIVmac239 Nef_core_ is rotated as a single rigid body in the orthorhombic crystal form with no significant changes in either main-chain or side-chain geometry, suggesting that the crystallographic Nef_core_ dimer interface is flexible and permissible to variations in crystal packing.

**Figure 9:**
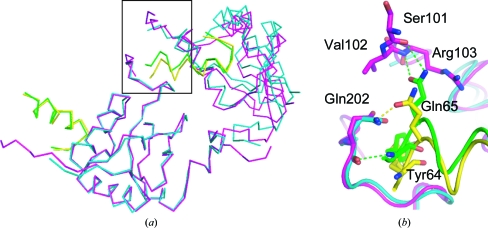
Variation in the crystal contact hydrogen-bond network. Overlay of the crystal-packing interface between two asymmetric units of the *P*2_1_2_1_2_1_ crystal lattice (SIVmac239 Nef is shown in magenta and TCRζ is shown in green) and two symmetry-related molecules (*y*, *x*, −*z*) and (1/2 + *y*, 1/2 − *x*, 1/4 + *z*) of the *P*4_3_2_1_2 crystal lattice (SIVmac239 Nef is shown in cyan and TCRζ is shown in yellow). Alignment was performed by least-squares methods using one SIVmac239 Nef–TCRζ polypeptide complex [at the bottom in (*a*)]. Hydrogen bonds present in the crystal lattices are represented by dashed lines and are colored green and yellow for the *P*2_1_2_1_2_1_ and *P*4_3_2_1_2 crystal forms, respectively.

Alternate crystal packing was also observed at the crystal con­tact of two asymmetric units in the orthorhombic crystal form and the corresponding symmetry-related molecules (*y*, *x*, −*z*) and (1/2 + *y*, 1/2 − *x*, 1/4 + *z*) in the tetragonal crystal form. The interface involves three proteins: SIVmac239 Nef_core_ and its bound TCRζ polypeptide ligand from the symmetry-related molecule (1/2 + *y*, 1/2 − *x*, 1/4 + *z*) and SIVmac239 Nef_core_ from the symmetry-related molecule (*y*, *x*, −*z*) (Fig. 9 ▶ *a*). Interestingly, the N-terminus of the TCRζ polypeptide abuts the neighboring SIVmac239 Nef_core_ protein, suggesting that the length of the N-terminal sequence of the TCRζ polypeptide ligand directs the space group in which the SIVmac239 Nef_core_–TCRζ polypeptide complex crystals grow. Superimposition of the TCRζ polypeptide helix from the symmetry-related molecule (1/2 + *y*, 1/2 − *x*, 1/4 + *z*) with its corresponding partner in the *P*2_1_2_1_2_1_ crystal form reveals that the neighboring SIVmac239 Nef_core_ protein is rotated ∼4.5° inwards towards the pseudo-fourfold symmetry axis in the *P*2_1_2_1_2_1_ crystal form (Fig. 9 ▶ *a*).

Accompanying the transformation is a possible reorganization of the hydrogen-bonding network at the crystal contact site. In the orthorhombic crystal form the TCRζ_A63–R80_ polypeptide forms a main-chain hydrogen bond to the neighboring SIVmac239 Nef_core_ protein between the main-chain amide of TCRζ Tyr64 and the side-chain carbonyl of Nef Gln202 (Fig. 9 ▶ *b*). TCRζ residue Gln65 additionally participates in hydrogen bonding to the main-chain amide and carbonyl of residues Arg103 and Val102, respectively, on its bound SIVmac239 Nef_core_ partner. Interestingly, this inter­action orders the proline-rich region in the N-terminus of the bound SIVmac239 Nef_core_ into a polyproline type II (PPII) helix as evidenced by the clearly resolved electron-density maps calculated from the *P*2_1_2_1_2_1_ crystal data for that region. This carries significant functional importance owing to the regulatory role that the PPII helix on HIV-1 Nef has been suggested to play in modulating kinase activity through its interaction with the SH3 domain of the kinase (Arold *et al.*, 1997 ▶; Lee *et al.*, 1996 ▶). The PPII helix was found to be dis­ordered in the unliganded HIV-1 Nef_core_ crystals and was only ordered in crystals containing the Fyn SH3 domain. Surprisingly, the hydrogen-bonding network between the TCRζ polypeptide and its bound SIVmac239 Nef_core_ partner is seemingly absent in the tetragonal crystal form; this explains the lack of electron density calculated from the *P*4_3_2_1_2 data for the N-terminus of SIVmac239 Nef_core_ since the PPII helix would no longer be expected to be ordered. Instead of participating in a side-chain–main-chain hydrogen bond with its bound partner, residue Gln65 in TCRζ is translocated in the tetragonal crystal form, bringing it into close enough proximity to residue Gln202 on the neighboring SIVmac239 Nef_core_ protein to participate in a side-chain–side-chain hydrogen bond. The main-chain–main-chain hydrogen bond between the TCRζ polypeptide and the neighboring SIVmac239 Nef_core_ protein is also lost in the rearranged *P*4_3_2_1_2 crystal contact interface.

Since the proposed hydrogen bond between Gln65 on TCRζ and Gln202 on SIVmac239 was formed by TCRζ and an adjacent SIVmac239 Nef_core_ protein in the crystal lattice and not its interacting SIVmac239 Nef_core_ partner, it is likely to be an artifact of crystallization that was necessary for proper lattice packing in the tetragonal crystal form. Curiously, the more physiologically relevant interaction of Gln65 on TCRζ with its bound SIVmac239 Nef_core_ partner was restored when the TCRζ polypeptide was truncated. The loss of the crystal contact hydrogen bond reduced the crystal symmetry to an orthorhombic crystal lattice that was subsequently prone to twinning. This was unexpected owing to the inclusion of a more complete TCRζ sequence in the tetragonal crystal and represents an interesting scenario in which a protein–ligand interaction was disrupted by a crystal contact interaction that permitted higher order crystal packing.

## 4. Conclusions

Crystal twinning can be induced by a number of perturbations, including heavy-metal soaking, ligand binding, selenomethionine substitution, flash-freezing and the introduction of point mutations (Parsons, 2003 ▶; Helliwell *et al.*, 2006 ▶). The structure determination of the two SIVmac239 Nef_core_–TCRζ polypeptide complexes provides a unique example of crystal twinning caused by the modification of peptide-ligand size. Truncation of the TCRζ polypeptide reduced the crystal symmetry from a tetragonal crystal system to an orthorhombic crystal system and introduced an NCS operation that only deviated slightly from the true fourfold symmetry axis. The pseudo-symmetry in the *P*2_1_2_1_2_1_ crystal made crystal growth highly susceptible to crystal twinning but serendipitously restored a physiologically relevant protein–ligand interaction at the crystal contact interface.

## Supplementary Material

PDB reference: Nef_core_–TCRζ_A63–R80_, 3ik5

PDB reference: Nef_core_–TCRζ_DP1_, 3ioz

Supplementary material file. DOI: 10.1107/S090744490904880X/yt5020sup1.pdf

## Figures and Tables

**Table 1:** Data-collection and refinement statistics (molecular replacement) Values in parentheses are for the highest resolution shell.

	Nef_core_–TCRζ_DP1_	Nef_core_–TCRζ_A63–R80_, crystal 1	Nef_core_–TCRζ_A63–R80_, crystal 2
Data collection			
Space group	*P*4_3_2_1_2	*P*2_1_2_1_2_1_	*P*2_1_2_1_2_1_
Unit-cell parameters			
*a* (Å)	51.64	47.19	47.42
*b* (Å)	51.64	47.24	47.42
*c* (Å)	189.45	182.99	183.52
α = β = γ (°)	90	90	90
Resolution (Å)	50–3.70 (3.83–3.70)	50–1.93 (2.02–1.93)	30–2.05 (2.12–2.05)
*R*_merge_	0.051 (0.393)	0.083 (0.513)	0.084 (0.498)
*I*/σ(*I*)	12.8 (2.1)	9.7 (2.4)	9.6 (2.5)
Completeness (%)	99.4 (100.0)	99.5 (98.8)	99.1 (96.5)
Redundancy	12.6 (12.7)	6.9 (5.9)	6.8 (5.2)
Twin fraction (*k*, *h*, −*l*)	N/A	0.500	0.426
Refinement			
Resolution (Å)	36–3.70		27–2.05
Total reflections	2923		25896
*R*_work_/*R*_free_	0.301/0.329		0.170/0.184
No. of atoms			
Protein	1054		2223
Water	N/A		116
NCS deviations (Å)	N/A		0.326
Average *B* factors (Å^2^)			
SIVmac239 Nef_core_	204.63		46.0
TCRζ polypeptide	219.51		49.2
Waters	N/A		45.2
R.m.s. deviations			
Bond lengths (Å)	0.003		0.003
Bond angles (°)	0.565		0.564
PDB code	3ioz		3ik5

## Footnotes

1 Supplementary material has been deposited in the IUCr electronic archive (Reference: YT5020). Services for accessing this material are described at the back of the journal.
