# Supplementary material for: Pseudo-merohedral twinning and noncrystallographic symmetry in orthorhombic crystals of SIVmac239 Nef core domain bound to different-length TCRζ fragments
Source: Acta Crystallogr D Biol Crystallogr. 2010 Jan 22;66(Pt 2):163–75. doi: 10.1107/S090744490904880X (PMC2815668; doi:10.1107/S090744490904880X)
Supplement: Supplementary file 1 [file d-66-00163-sup1.pdf]

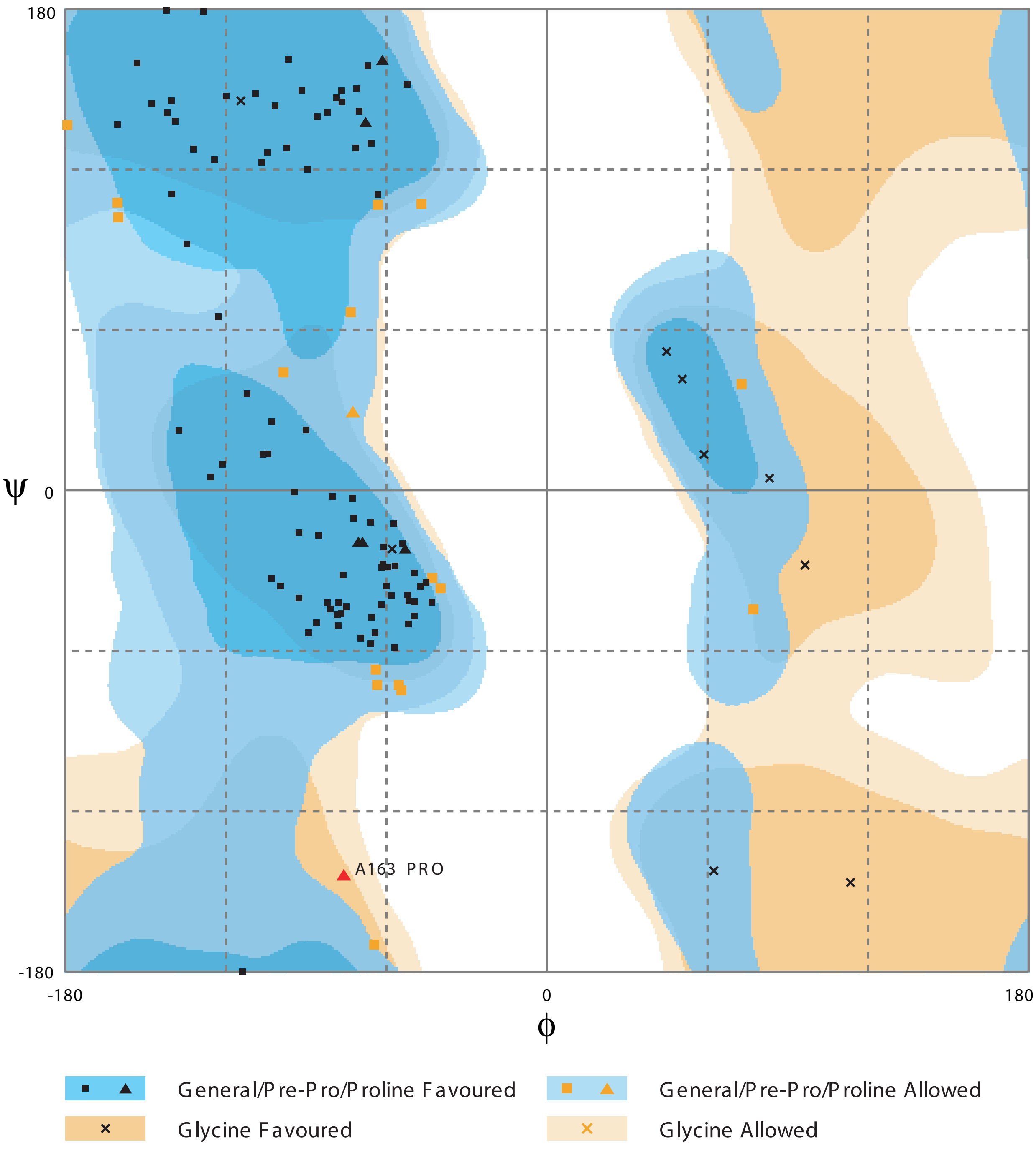

Number of residues in favoured region (~98.0% expected) : 101 (84.9%)
Number of residues in allowed region (~2.0% expected) : 17 (14.3%)
Number of residues in outlier region : 1 (0.8%)

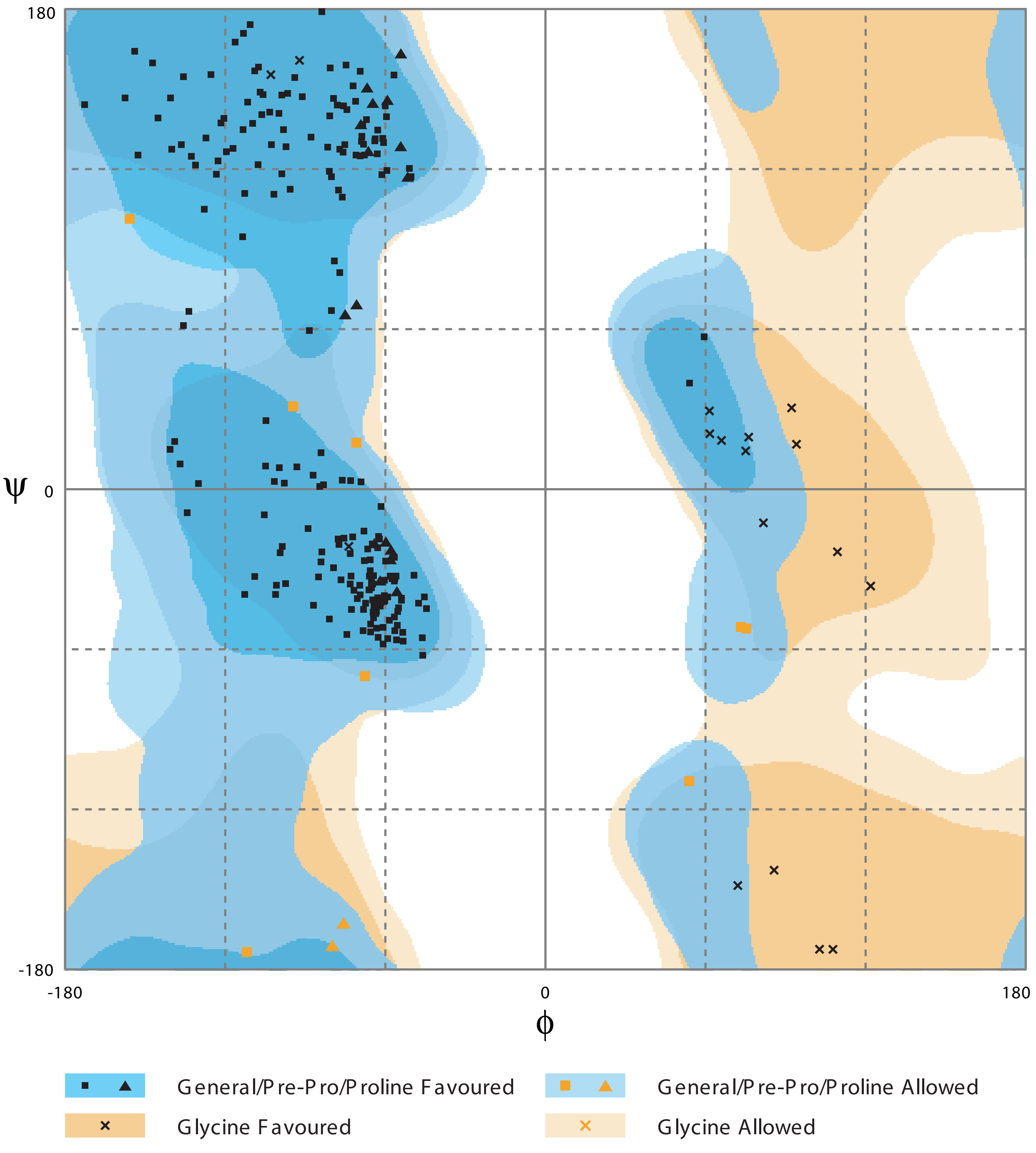

Number of residues in favoured region (~98.0% expected) : 245 (96.1%)
Number of residues in allowed region (~2.0% expected) : 10 (3.9%)
Number of residues in outlier region : 0 (0.0%)
